# Early postoperative interventional ASD-closure for severe atrial right to left shunt in a neonate with common arterial trunk

**DOI:** 10.3325/cmj.2013.54.394

**Published:** 2013-08

**Authors:** Daniel Dilber, Andreas Eicken, John Hess

**Affiliations:** 1 Division of Pediatric Cardiology, Department of Pediatrics, University Hospital Zagreb at the Zagreb University School of Medicine, Zagreb, Croatia; 2 Departement of Pediatric Cardiology and Congenital Heart Disease, German Heart Center, Technical University Munich, Germany

## Abstract

Although closure of an atrial septal defect (ASD II) with an occluding device in the first year of life is not a routine procedure, it is a feasible treatment, even in neonates. Case reports on the off-label use of Amplatzer devices have been repeatedly published, but there are no reports on using the Amplatzer Duct Occluder (ADO) to close an atrial septal defect in a neonate. We report on a successful catheter closure of an ASD II with ADO in a severely cyanotic neonate, seven days after surgical repair of common arterial trunk. Due to progressive cyanosis and clinical signs of right ventricular failure, which developed after common arterial trunk repair, the neonate underwent cardiac catheterization. Diastolic filling impairment of the right ventricle (right ventricle hypertrophy, pulmonary regurgitation, and residual right ventricle outflow tract obstruction) was thought to be the cause of impaired right ventricle diastolic filling, resulting in the right-to-left shunt at the atrial level. Under transesophageal echocardiographic guidance, ADO was delivered through a 5 French sheath into the atrial septal defect. Amplatzer duct occluder closed the defect and proved to be stable in position after disconnection. During the procedure, the child was stable and then transferred to the intensive care unit with significantly improved oxygen saturation. This is the first report on placing a duct occluder in the atrial septal position, which is a novel procedure for-small neonates.

Catheter interventional device closure of an atrial septal defect is the first line treatment in many centers and is routinely performed in patients aged 4-6 years. Device closure of an atrial septal defect (ASD) in the first year of life is not a routine procedure, because of known problems (1), but is feasible (2) even in neonates (3). Case reports on the off-label use of Amplatzer devices have been repeatedly published (4). The Amplatzer duct occluder was initially developed to close an open ductus arteriosus (5). To our knowledge, there are no reports on using the Amplatzer Duct Occluder 2 (ADO) to close an ASD II in a neonate. We successfully performed this procedure in a symptomatic neonate shortly after surgical repair of a complex congenital heart disease.

## METHODS

**Figure 1:**
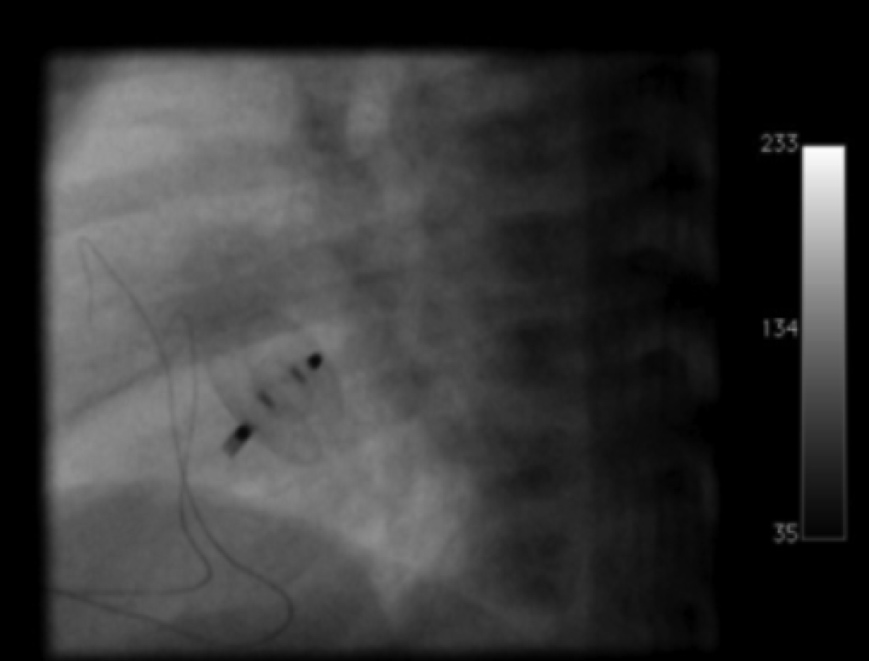
Amplatzer duct occluder in the atrial septal position

The patient was a female neonate, born after 36 weeks of uneventful pregnancy by spontaneous vaginal delivery (weight 3100 g). Fetal echocardiography revealed the prenatal diagnosis of common arterial trunk. By post-natal echocardiography, we confirmed the diagnosis of common arterial trunk, type A1 (6) with a dysplastic tricuspid truncal valve overriding the ventricular septum, with moderate truncal valve stenosis (gradient 25 mm Hg) and insufficiency (grade II), right-sided aortic arch, and a small atrial septal defect type secundum with a left-to-right shunt. Eleven days after birth, she underwent surgical repair. After excision of the pulmonary arteries from the trunk, the resulting defect was closed using glutar-aldehyde-treated pericardium, the ventricular septal defect was enlarged and then closed with a Gore-Tex patch, and continuity from the right ventricle to the pulmonary arteries was accomplished with pericardium and a Gore-Tex patch. The atrial septal defect was left open as a solution to preserve left ventricle output in case of cardiac insufficiency in early postoperative period. The patient was weaned from cardiopulmonary bypass without difficulties and transferred to the intensive care unit, where she received mechanical ventilation and mild inotropic support. During the following days, the child presented with progressive cyanosis and clinical signs of right ventricular failure. Additionally, runs of junctional ectopic atrial tachycardias occurred. Echocardiography demonstrated a right-to-left shunt across the ASD, as well as an elevated right ventricular pressure, with a peak systolic Doppler flow of 3.4 m per second at the level of the tricuspid valve. The right ventricle outflow tract (RVOT) was obstructed by the protrusion of the ventricular septal defect (VSD) patch to the right, probably due to moderate aortic insufficiency directed at the patch. Cyanosis was progressive and not responsive to ventilation with inhaled nitric oxide and 100% oxygen. On the 7th postoperative day, the child underwent cardiac catheterization. Half systemic pressures were found in the right ventricle, with a peak systolic gradient of 20 mm Hg in the RVOT. The pulmonary venous oxygen saturations were 99%, however, the arterial oxygen saturation was only 69%. Angiography revealed a mild RVOT obstruction, severe pulmonary regurgitation, mild tricuspid regurgitation, small residual VSD, a moderate truncal valve regurgitation, and severe right-to-left shunt through the ASD. Upon a temporary test occlusion of the ASD with a regular Berman 5 F balloon catheter, arterial oxygen saturation immediately rose from 69% to 92%. After twenty minutes, the right atrial pressures slightly rose from 8/8/7 mm Hg to 13/12/11 mm Hg. Diastolic filling impairment of the right ventricle (right ventricle hypertrophy, pulmonary regurgitation, and residual RVOT obstruction) likely resulted in impaired right ventricle diastolic filling, which finally led to the right-to-left shunt at the atrial level. Due to the small patient size, device closure with a regular ASD-closure device did not seem appropriate. For the same reason, balloon sizing of the ASD was not done. Under transesophageal echocardiographic guidance an Amplatzer Duct Occluder 2 6/4 (Medical Corporation, North Plymouth, MN, USA) was delivered through a 5 French sheath into the atrial septal defect (Figure 1).

## RESULTS

Under transesophageal guidance, ADO 2 was safely delivered into the atrial septal defect. It closed the defect and proved to be stable in position after disconnection. During the procedure, the child was stable and then transferred to the intensive care unit with significantly improved oxygen saturation. On the following day, the infant was successfully extubated and the inotropic support could be stopped. In the next few days, she suffered from arrhythmias (junctional ectopic tachycardia), which were treated by atrial pacing synchronized to the R wave. After 23 days in the intensive care unit, the patient was transferred to the in-patient cardiology ward. She had no residual shunt at the level of the atrial septum, moderate insufficiency of the aortic valve without stenosis, no significant stenosis of the RVOT, and normal pressures in the right ventricle. Two weeks later, the child was discharged home. It was estimated that in the near future the patient would probably need a valved conduit between the right ventricle and the pulmonary artery, or mechanical valve. However, repeated surgical intervention in this severely cyanotic patient was believed to be highly risky, which led to the decision to close the ASD II by catheter intervention, which clearly improved the clinical situation. The child was followed up in the echocardiography unit and the last examination performed two years after the procedure showed satisfactory findings, adequate position of ADO, with no residual shunt of the level of atrial septum and no residual ventricular septal defect.

## DISCUSSION

This report shows that early postoperative catheter interventional ASD occlusion in a critically ill, cyanotic neonate after surgical repair of common arterial trunk is feasible with ADO 2. The patient experienced instant improvement in oxygen saturation and was completely weaned off mechanical ventilation by the following day. However, it was estimated that in the near future a valved conduit between the right ventricle and the pulmonary artery or mechanical valve implantation will be needed. Since she was severely cyanotic and a repeated surgical intervention was considered risky, we opted to close the ASD II by catheter intervention. Device closure with a regular ASD-closure device did not seem appropriate due to the small size of the patient. For the same reason, balloon sizing of the ASD was not done. We closed the defect successfully with ADO 2, which may additionally be delivered through a smaller sheath, such as a 4 F sheath. Under transesophageal guidance ADO 2 was safely delivered into the atrial septal defect. It successfully closed the defect and proved to be stable in position after disconnection. In conclusion, our case report shows that effective closure of an atrial septal defect by “off-label” use of ADO 2 is feasible in small children, even in the early post operative period.
